# Report of Dental Educational Council of America

**Published:** 1918-11

**Authors:** H. L. Banzhap


### REPORT OF DENTAL EDUCATIONAL COUNCIL OF AMERICA

BY H. L. BANZHAF, D. D. S.

Minimum requirements for Class "A" dental schools, adopted by the Dental Educational Council of America, at Louisville, Ky., July 24, 1918, revised at New York City, October 22, 1917, and March 26, 1918.

### ADMINISTRATIVE POLICY

Section 1. (a) The administrative policy of the college must be satisfactory to the Dental Educational Council of America. The Dean or other executive officer must hold and have authority to carry out fair ideals of dental education.

(b) The value of the building and equipment (grounds excluded) must be equal to at least $300.00 for every student enrolled.

(c) The school must have facilities and equipment for at least twenty-five students in each class.

### ENTRANCE REQUIREMENTS

Section 2. (a) The requirements for entrance shall consist of graduation from an accredited high school or academy which requires for graduation not less than fifteen units of high school work obtained in a four-year course beyond the eighth grade of the elementary school. No conditions on the foregoing entrance requirement shall be allowed.

(b) An accredited high school is defined as one which is accredited as a four-year high school by the United States Bureau of Education, or by a university which is a member of the Association of American Universities or by the State University of the state in which the high school is located.

(c) In the case of an applicant who is not a graduate from a high school or academy, as defined above, the full equivalent of such education in each individual case must be established and attested to by the highest public educational officer of the state in which is located the dental college which the applicant seeks to enter.

(d) The entrance credentials of each student enrolled must be kept on file and open to general inspection until after graduation. Not later than sixty days after the opening of school the Dean shall send to the secretary of the Dental Educational Council and to the secretary of the local state board of dental examiners a complete list of the students enrolled, together with a sworn statement that each student is possessed of the entrance qualifications outlined above.

(e) Students with two full years credit from Class ''A'' medical schools, approved by the American Medical Association, may be admitted to the sophomore class. No other advanced credit in time may be given in any other case than above specified. No special students shall be accepted unless they are in possession of the entrance requirements specified above.

(f) The foregoing regulations apply to all students including those from foreign countries, and regardless of where the applicant expects to practice his profession.

### COURSE OF STUDY

Section 3. (a) Beginning with the session 1917-18 the course must be four years in length, each year to consist of thirty-two weeks and six days in each week.

No degrees other than Doctor of Dental Surgery, Doctor of Dental Medicine, or Doctor of Dental Science may be given. Dental subjects may be taught throughout the entire four years. Schools that offer a three-year course with one year of college work as a prerequisite shall not be regarded as satisfactory.

(b) The school must offer a course of at least 4,400 hours laboratory and didactic instruction.

(c) The minimum hours devoted to each branch shall be as follows:

| | |
|---|---|
| Operative and Clinical Dentistry | 1300 |
| Prosthetic Technics | 384 |
| Crown and Bridge Technics | 320 |
| Operative Technics | 160 |
| Oral Hygiene | 32 |
| Dental Anatomy | 96 |
| Orthodontia | 96 |
| Oral Surgery | 96 |
| Physics, Biology, or both | 192 |
| Chemistry (Inorganic—Organic—Physiological—Metallurgy) | 320 |
| Technical Drawing | 48 |
| Anatomy | 320 |
| Histology | 128 |
| Pathology (General and Dental) | 128 |
| Materia Medica | 64 |
| Bacteriology | 128 |
| Physiology | 128 |
| Dental Rhetoric | 96 |
| Physical Diagnosis, Anesthesia | 32 |
| Radiology | 32 |
| Jurisprudence, Dental History, Ethics, Economics | 32 |
| Additions to above, or other subjects | 268 |
| Total | 4400 |

### TEACHING FACILITIES

Section 4. (a) The classes in dentistry must be taught separately from the classes in any of the other departments, if the dental school in question is part of a university.

(b) The patronage of the infirmary clinic must be such as to give each student at least 150 operations in fillings (gold, inlay, amalgam, cement, root fillings, etc.) prosthetic work, and orthodontia. Treatments preparatory to the above, and cases of exodontia must not be included in the number stated above.

(c) In the anatomical laboratory not more than eight students, working in pairs, may be assigned to one cadaver for a complete dissection.

(d) Every twenty students working in the infirmary at any given time must have the undivided services of at least one demonstrator.

(e) Every thirty students working in the scientific laboratories must have the undivided services of at least one instructor.

(f) Every forty students working in the technic laboratories must have the undivided services of at least one instructor.

(g) No persons except those holding the D. D. S., M. D., or bachelor's degree or equivalent, or who hold a license to practice dentistry, shall be employed as instructors.

### LABORATORIES AND OTHER FACILITIES

Section 5. (a) The school must be possessed of the following number of laboratories and class rooms, equipped in the following manner:

(b) One chemical laboratory equipped to adequately teach qualitative, quantitative, general inorganic and organic chemistry and physiological chemistry.

(c) One microscopical laboratory equipped with sufficient high power microscopes so that each student may be possessed of the use of a microscope when he is working in the laboratory.

(d) Sufficient class rooms—at least one of which must be equipped with lantern for projection.

(e) Sufficient technic laboratories, so that each student in attendance is provided with an individual place for laboratory work.

(f) A dental infirmary, equipped with a sufficient number of dental chairs to adequately serve the senior class. An efficient equipment for sterilizing students' instruments must be provided.

(g) An X-ray outfit for use in conjunction with the dental infirmary.

(h) A dental library constantly available to the students, which shall have at least twice the number of volumes as there are students enrolled in the school.

### STATE BOARD RECORD

Section 6. The college must not have more than twenty-five per cent. failures before the various state boards more than two years in succession.

### ATTENDANCE

Section 7. The record of attendance required of students must not be less than eighty-five per cent. for each year.

### PROMOTION OF STUDENTS

Section 8. (a) A student who has incomplete course conditions, or failures, in 60 per cent. of his course for any semester shall be dropped.

(b) A student may not be promoted if he has incomplete conditions or failures in more than 20 per cent. of the course of any year.

(c) A student who fails to remove a condition or failure within twelve months from the time it was incurred shall automatically be dropped from the college.

(d) An incomplete course is one that has not been completed because of illness or other personal emergency.

PASSING MARK, CONDITIONS AND FAILURES

Section 9. (a) The passing mark shall be 75 per cent.

(b) A grade between 60 and 74 per cent. is defined as a condition.

(c) A grade below 60 per cent. is defined as a failure.

(d) A condition may be removed by examination.

(e) A failure may not be removed except by repetition of the course in part or entirely, *i. e.*, by additional work under instruction approved by the Dean or the Professor in charge of the subject.

(f) A condition which is not removed within thirty days of the opening of the next year, automatically becomes a failure and can then only be removed by a repetition of the course.

(g) If a college grades by letters it shall state (publish) definitely the percentage range value of each letter used in designation of standings.

CLASS "B" DENTAL SCHOOL DEFINED

Schools which in certain particulars do not meet the requirements for Class "A," but may become eligible for Class "A" without complete reorganization.

CLASS "C" DENTAL SCHOOL DEFINED

Schools which could not meet the requirements for Class "A" without very extensive improvements and a complete reorganization. Class "C" schools shall be considered "not well recognized dental schools."

TRANSFER OF DENTAL STUDENTS

Students of dental celleges which have been well recognized but lost this recognition may transfer to well recognized dental colleges, if acceptable to these colleges. Such students may be accepted as far as entrance requirements are concerned on the requirements of the college in which the student began the study of dentistry.

Under Section 1, "Administrative Policy," another subdivision was added as follows:

"The conduct of a dental school for profit to individuals or a corporation does not meet the standard of fair ideals, as interpreted by the Dental Educational Council of America."

Under "Entrance Requirements," Section 2 (a), the following amendment was made: In the fourth line of the paragraph the following words were stricken out, "beyond the eighth grade of the elementary school," so that when amended this section reads:

"Section 2. (a) The requirements for entrance shall consist of graduation from an accredited high school or academy which required for graduation not less than fifteen units of high school work obtained in a four-year course. No conditions on the foregoing entrance requirement shall be allowed."

Under Section 7. "Attendance," the following words were added:

"Attendance shall be counted from the close of registration," so that when amended this section shall read:

"Section 7. The record of attendance required of students must not be less than eighty-five per cent. for each year. Attendance shall be counted from the close of registration."

Under the head of "Promotion of Students," Section 8 (a) following amendment was adopted: Strike out the word "course" as it occurs in the first line of the paragraph, and substitute therefor the word "courses;" strike out the word "conditions;" strike out the word "60 per cent." and substitute therefor the word "40 per cent.," and add to the end of the paragraph the words "from his class," so that when amended Section 8 (a) shall read as follows:

"Section 8. (a) A student who has incomplete courses or failures in 40 per cent. of his course for any semester shall be dropped from his class."

Under Section 8 (b), the word "incomplete" was struck out, so that it reads:

"Section 8. (b) A student may not be promoted if he has conditions or failures in more than 20 per cent. of the course of any year.

The following classification of dental schools was adopted:

CLASS "A"

University of Southern California, College of Dentistry, Los Angeles.

University of California, College of Dentistry, San Francisco.

Northwestern University Dental School, Chicago, Ill.

University of Illinois, College of Dentistry, Chicago, Ill.

University of Iowa, College of Dentistry, Iowa City.

University of Michigan, College of Dentistry, Ann Arbor.

University of Minnesota, College of Dentistry, Minneapolis.

Creighton University Dental School, Omaha, Nebraska.

Ohio State University, College of Dentistry, Columbus.

North Pacific Dental College, Portland, Oregon.

University of Pittsburgh, College of Dentistry, Pittsburgh, Pa.

The Thomas W. Evans Museum and Dental Institute, University of Pennsylvania, Philadelphia, Pa.

Medical College of Virginia, School of Dentistry, Richmond, Va.

Marquette University, College of Dentistry, Milwaukee, Wis.

TENTATIVE CLASS "A"

Harvard Dental School, Boston, Mass., and Tufts Dental College, Boston, Mass.

Classification withheld until these schools meet the Council's requirements respecting the evaluation of entrance credentials. When this is done they automatically pass into Class "A."

CLASS "B"

Colorado College of Dental Surgery, Denver, Colo.

Georgetown University, School of Dentistry, Washington, D. C.

Howard University Dental School, Washington, D. C.

Atlanta-Southern Dental College, Atlanta, Ga.

Louisville University, College of Dentistry, Louisville, Ky.

Chicago College of Dental Surgery, Chicago, Ill.

Indiana Dental College, Indianapolis, Indiana.

Loyola University, School of Dentistry, New Orleans, La.

Baltimore College of Dental Surgery, Baltimore, Md.

University of Maryland, Dental Department, Baltimore, Md.

St. Louis University, College of Dentistry, St. Louis.

Washington University Dental School, St. Louis.

Kansas City Dental College, Kansas City, Mo.

Western Dental College, Kansas City, Mo.

University of Buffalo, Dental Department, Buffalo, N. Y.

New York College of Dentistry, New York.

College of Dental and Oral Surgery of New York.

Western Reserve University Dental School, Cleveland, Ohio.

Ohio College of Dental Surgery, Cincinnati, Ohio.

Philadelphia Dental College, Philadelphia, Pa.

Vanderbilt University, School of Dentistry, Nashville, Tenn.

University of Tennessee, College of Dentistry, Memphis, Tenn.

Meharry Dental College, Nashville, Tenn.

College of Physicians and Surgeons, Dental Department, San Francisco, California.

George Washington University Dental School, Washington, D. C.

CLASS "C"

Lincoln Dental College, Lincoln, Neb.

College of Jersey City, N. J.

Cincinnati College of Dental Surgery, Cincinnati, O.

State Dental College, Dallas, Texas.

Texas Dental College, Houston, Texas.

Respectfully submitted.—HENRY L. BANZHAF, *Secretary-Treasurer*.

